# The effects of PPO activity on the proteome of ingested red clover and implications for improving the nutrition of grazing cattle

**DOI:** 10.1016/j.jprot.2016.04.023

**Published:** 2016-06-01

**Authors:** E.H. Hart, L.A. Onime, T.E. Davies, R.M. Morphew, A.H. Kingston-Smith

**Affiliations:** Institute of Biological, Environmental and Rural Sciences (IBERS), Aberystwyth University, Penglais, Aberystwyth SY23 3FG, UK

**Keywords:** PPO, Polyphenol oxidase, Red clover, Protein complexing, Rumen, Chloroplast

## Abstract

Increasing the rumen-stable protein content of feed would lead to improved nitrogen utilisation in cattle, and less nitrogenous waste. Red clover (*Trifolium pratense* L.) is a high protein ruminant feed containing high polyphenol oxidase (PPO) activity. PPO mediated protein-quinone binding has been linked to protecting plant proteins from proteolysis. To explore the mechanism underlying the effect of PPO on protein protection in fresh forage feeds, proteomic components of feed down-boli produced from wild-type red clover and a low PPO mutant, at point of ingestion and after 4 h in vitro incubation with rumen inoculum were analysed. Significant differences in proteomic profiles between wild-type and mutant red clover were determined after 4 h incubation, with over 50% less spots in mutant than wild-type proteomes, indicating decreased proteolysis in the latter. Protein identifications revealed preferentially retained proteins localised within the chloroplast, suggesting that PPO mediated protection in the wild-type operates due to the proximity of target proteins to the enzyme and substrates, either diffusing into this compartment from the vacuole or are present in the chloroplast. This increased understanding of protein targets of PPO indicates that wider exploitation of the trait could contribute to increased protein use efficiency in grazing cattle.

**Biological significance:**

One of the main challenges for sustainable livestock farming is improving capture of dietary nitrogen by ruminants. Typically up to 70% of ingested protein-N is excreted representing a loss of productivity potential and a serious environmental problem in terms of nitrogenous pollution of lands and water. Identification of key characteristics of rumen-protected protein will deliver target traits for selection in forage breeding programmes. The chloroplastic enzyme PPO catalyzes the oxidation of phenols to quinones, which react with protein. Little is currently known about the intracellular protein targets of the products of PPO activity or the mechanism underlying protein complexing, including whether there is any specificity to the reaction. Here we have determined significant differences in the proteomes of freshly ingested down boli corresponding to the presence or absence of active PPO. These results show that in the presence of PPO the forage protein is less amenable to proteolysis and provide the novel information that the protected proteins are putatively chloroplastically located. These data also contribute to a growing evidence base that a chloroplastic PPO substrate exists in red clover in addition to the currently known vacuolar substrates.

## Highlights

• PPO has potential for protein protection in forage feeding systems.
• Proteome profile depended on presence or absence of PPO.
• Preferentially retained proteins were chloroplast localised.

## Graphical abstract

**Figure:**
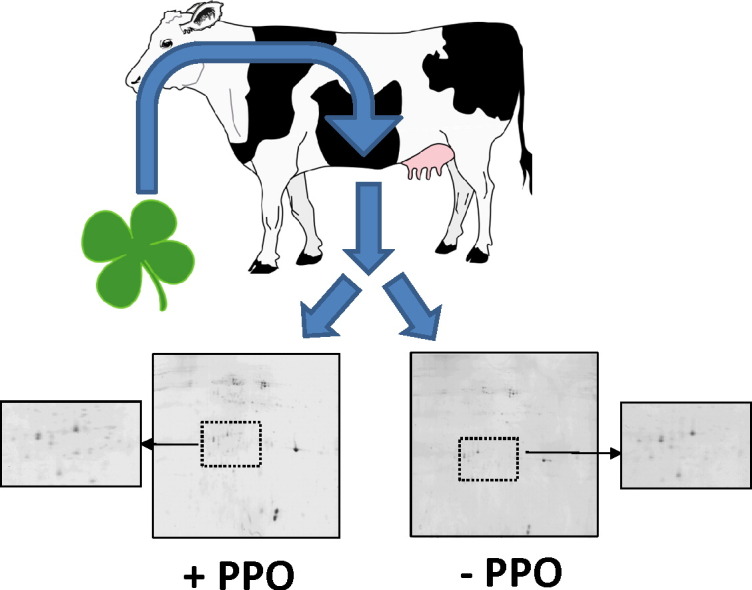

## 1 Introduction

The rapid breakdown of proteins from forage feed and the inefficient capture of the breakdown products by rumen microbiota is the foremost source of nitrogen loss from cattle production systems [1], [2]. The inadequate utilisation of dietary protein by ruminants results in high economic losses to farmers and has huge negative implications for the environment in ruminant agricultural systems [3]. Dietary protein from forage materials is degraded by proteolytic rumen micro-organisms [4] and in fresh forage feeding systems by endogenous plant proteases, particularly during the initial phase of protein degradation [5], [6], [7], [8]. As these plant enzymes could make a significant contribution to rumen function [6], [7], [8] understanding forage based-mechanisms by which to manipulate rates of protein breakdown in ingested feed would be advantageous in terms of mitigating the environmental impact of N-deposition resulting from livestock farming systems.

Red clover (*Trifolium pratense* L.) provides a high-protein feed for grazing livestock and during conservation is prone to lower levels of protein degradation compared with other legume feed sources such as alfalfa [9]. Despite alfalfa and red clover being of similar protein content [10], between 44 and 87% of protein is degraded during ensilage of alfalfa whereas only 7–40% of protein is degraded in red clover [11]. This reduction in the extent of postharvest proteolysis in red clover is due to the activity of polyphenol oxidase (PPO) [12]. PPO is a copper metall*o*-protein that catalyses the oxidation of endogenous phenols to quinones in the presence of oxygen [13]. These reactive *o*-quinones can then form covalent bonds with the nucleophilic groups of proteins, such as sulfhydryl, amine, amide, indole or imidazole groups due to their electrophilic nature [14], [15]. The digestibility of these complexed plant proteins is greatly reduced, and consequently the amount of non-degraded dietary protein flow to the small intestine is increased, which is advantageous to the efficiency of a ruminant production system [16]. PPO occurs in either an active or a latent state. In red clover PPO is typically found in its latent form, with the active enzyme accounting for approximately 20% of the total PPO [17]. Activation and activity of latent PPO is prevented by the differential compartmentalisation of the enzyme which is present in the chloroplast and the known substrates, phaselic acid and clovamide, which are reputed to be present in the vacuole [18], [19], [20]. Proposed roles for PPO include defence mechanisms, oxygen regulation, electron transport and involvement in the Mehler reaction [21], [22], [23], [24] plus a complex involvement in plant–microbe interactions [23], [24].

PPO has been well studied in red clover in relation to production of silage feeds for ruminants [25-27], with recent studies focusing on genomic and transcriptomic analysis of the members of the PPO multigene family [28], [29]. However, little is currently known about the specificity of protein targets involved in quinone-protein complexing reactions mediated by PPO which potentially limits our ability to increase protein stability in forages. Identification of core features of the endogenous protein targets would allow quality traits to be included in selective breeding programmes for forage crop improvement. Ruminants are extremely inefficient in their use of forage protein. Typically up to 70% of the ingested protein is not incorporated into milk or meat product but is excreted leading to widespread environmental pollution and availability of substrates for N_2_O production. In real terms this equates to a loss of approximately 150 g N per head of cattle per day, or 54.6 kg per head per year [30]. Hence, even an apparently insignificant increase (i.e. ~ 5%) in protein stability in the rumen would result in a significant improvement to protein use efficiency; the increased availability of protein for uptake by the animal in post-rumen digestion would decrease the need for on farm supplemental protein feeds and thereby decrease production of nitrogenous wastes.

In order to understand the potential benefit that could be conferred by PPO activity within the rumen system in the grazing context we have taken a proteomic led approach to explore the effect of PPO on protein complexing in ingested forage. Comparisons of 2D gels were made based on the null hypothesis that protein profiles from wild-type and mutant samples would be the same if all protein is degraded to the same extent regardless of the presence or absence of PPO. We have compared the proteomes formed from wild type red clover and a naturally occurring mutant line, which contains low PPO activity through a lack of PPO4 expression in the leaves [17], both in terms of the immediate effect of masticative ingestion and after fermentation in the presence of a rumen microbial inoculum.

## 2 Materials and methods

All experimentation involving animals was conducted in accordance with the U.K. Animals (Scientific Procedures) Act 1986. Six Holstein-Friesian non-lactating dairy cows, each fitted with permanent rumen cannula had free access to a perennial ryegrass pasture diet prior to commencement of the experiment.

### 2.1 Plant materials

Freshly cut plant material from red clover wild type (*Trifolium pratense* cv *Milvus*; WT, Aa 4381) and a low PPO mutant (M) lacking expression of PPO4 (Aa 4521) [13], [17] were obtained from adjacent plots at Trawscoed Research Farm, Aberystwyth, which had been cut and fertilised 4 weeks prior to harvesting. Forage was harvested from plots with a Haldrup 1500 plot harvester (J. Haldrup a/s, Løgstør, Denmark) and cut to 5 cm above the soil to allow regrowth.

### 2.2 Preparation of rumen fluid inocula

Approximately 200 ml rumen fluid from each cow was removed and combined and passed through two layers of muslin. This was prepared as a 10% inoculum with Van Soest buffer for use in in vitro fermentations as described previously [31] and kept at 39 °C until required.

### 2.3 Collection of feed down boli

Food was removed from animals 16 h prior to offering the test forages. Each cow was offered fresh forage in a randomised cross-over design. Water was available ad libitum throughout the experiment. Immediately before bolus collection rumen contents were emptied from each animal and kept warm until replaced. Newly ingested down boli were collected at the oesophageal junction, accessed from a rumen cannula. The first three boli from each animal and treatment were discarded to avoid contamination from previously ingested forage. Boli numbers four and five were retained for analysis.

### 2.4 Bolus incubation and sample preparation

**Fig. 1:**
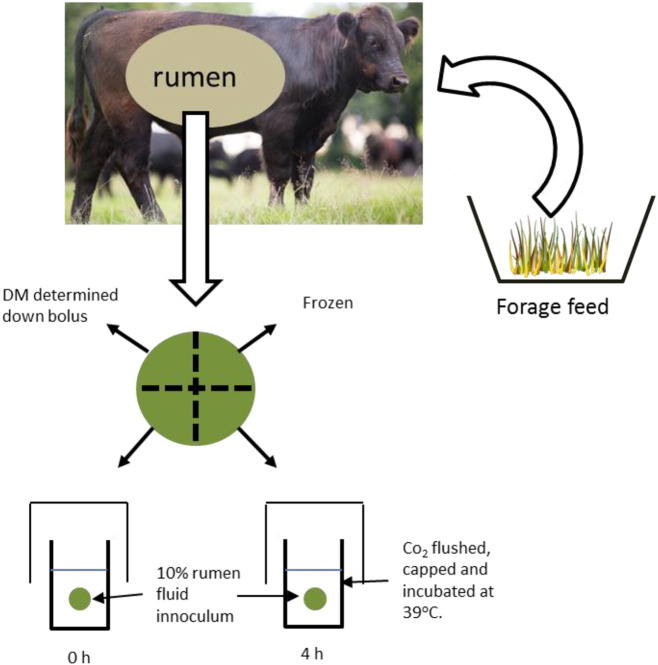
Diagrammatic representation of the collection and in vitro incubation of feed down boli.

Each retained bolus was individually placed in muslin and sealed with an elastic band. The boli were rinsed in water for 45 s (boli were immersed five times and gently squeezed while still in the water, the whole process repeated a total of five times). Each bolus was then drained, divided into quarters and weighed. One quarter was immediately frozen in liquid nitrogen as an untreated sample and a second quarter was used for dry matter (DM) determination, conducted by drying samples to a constant weight at 105 °C prior to N determination by the Dumas method [32]. The remaining two quarters were placed individually into each of two 250 ml Duran bottles containing 100 ml of the 10% rumen fluid inoculum. One quarter bolus was removed immediately from one bottle (0 h sample) while the other was flushed with CO_2_ and capped tightly for 4 h incubation at 39 °C (Fig. 1). This time period was chosen to specifically assess the effect of PPO during the initial stages of colonisation and feed degradation in the rumen [33], [34], [35]. After exposure to rumen fluid the boli were removed from the bottles, drained in a sieve and rinsed with 100 ml deionised water before they were lyophilised to a constant weight and stored at − 20 °C prior to protein extraction. At both 0 and 4 h post incubation a 4 ml subsample of buffered rumen fluid was also removed from the bottles and preserved with 1 ml orthophosphoric acid containing 20 mM 2-ethyl butyric acid and stored at 4 °C prior to determination and quantification of volatile fatty acids (VFA) by GC against known standards as described previously [36].

### 2.5 PPO quantitation

PPO activity was determined spectrophotometrically by measuring the initial linear rate of change in absorption at λ 420 nm according to the method of [17] on subsamples of each boli. One PPO activity unit was determined as a change of 0.01 in the absorbance per min, and was expressed as per gram of fresh weight of the sample (U/g FW). The active pool of PPO was determined by adding 20 μl of desalted protein fraction to a cuvette containing 1.135 ml of a reaction buffer (88 ml of McIlvaine buffer (pH 7), containing 1.2 ml of 0.15 mM copper sulphate and 1.6 ml of water) with the reaction initiated by the addition of 10 mM methylcatechol as the substrate. Total PPO activity (active plus latent pools) was determined as described above except that 0.26% SDS (w/v) was also included in the reaction mixture. Significant differences (*P* > 0.05) between PPO levels were determined by ANOVA analysis using Genstat, [37].

### 2.6 Protein sample preparation for 2DE

Protein was extracted from 0.5 g DW of the bolus samples recovered after 0 and 4 h incubation. Samples were ground twice into a fine powder under liquid nitrogen and then incubated overnight at − 20 °C in 5 ml of extraction buffer; 20% TCA, 1% phosphotungstic acid (PTA), and 0.2% DTT in ice cold acetone. Extracts were then centrifuged at 21,000 ×* g* at 4 °C for 30 min and the resulting pellet washed with 0.2% DTT in cold acetone and incubated at -20 °C for 1 h. This wash process was repeated twice before the protein extracts were air dried and re-suspended in 8 M urea, 2 M thiourea, 4% CHAPS, 50 mM DTT and 0.8% pharmalytes pH 3–10 (Amersham, Little Chalfont, Buckinghamshire, U.K.) according to [38]. Quantification of protein content in prepared samples was conducted by the method of [39] with reference to a BSA standard.

### 2.7 2DE and image analysis

To determine whether the protein distribution was different between genotype and/or treatment times an equal volume of protein was applied for each sample. Changes were determined in relative abundance of individual spots. A total of 150 μg of protein from the re-suspended protein extracts were passively rehydrated overnight on 7 cm non-linear IPG strips (pH 3–10) and then focused to 10,000 VH using the Protean IEF cell (BioRad Ltd, Hemel Hempsted,UK) as described previously [40]. The use of 7 cm strips is consistent with previous studies into the plant proteome [41], [42], [43]. Equilibration of each IPG strip was conducted for 12 min in 2.5 ml equilibration buffer (50 mM Tris-HCl pH 8.8, 6 M urea, 30% glycerol v/v and 2% SDS w/v), with the presence of DTT (Melford, Ipswich, U.K.) at 10 mg/ml followed by a second equilibration with IAA (iodoacetamide) (Sigma, Gillingham, U.K.) at 25 mg/ml. Proteins were separated in the second dimension using the Mini Protean system (BioRad Ltd, Hemel Hempsted, UK) at 180 V using 14% T, 3.3% C polyacrylamide gels. Gels were visualised via Coomassie blue staining (PhastGel Blue R, Amersham Biosciences, U.K.) and images captured using a GS-800 calibrated densitometer (Biorad Ltd, Hemel Hempsted, U.K.) and analysed using Progenesis (PG220 v.2006. Nonlinear Dynamics, Newcastle upon Tyne, UK). Analysis was performed on three biological replicates using normalised spot volumes to identify spots showing a ± 2 fold change in protein abundance. Protein abundances were further analysed using a two way ANOVA (Genstat) [37].

### 2.8 Protein identification and database searching

Spots of interest, determined as increase or decrease in protein abundance, unique or landmark were subjected to trypsin digestion according to the method of [44] with slight modification [45]. Mass spectrometry was performed using an Agilent 6550 QTOF LC MS/MS (Agilent, Cheshire, UK).

Peptides were analysed using Mass Hunter software (Agilent, UK) and tandem mass spectra (MS/MS) queries were performed using the MASCOT database search engine v2.1 (Matrix Science, London, U.K.) [46] on the Swiss-Prot database. Searches were restricted to the Viridiplantae taxonomy with trypsin specificity (one missed cleavage allowed), the tolerances for peptide identification were set as 0.3 Da. Fixed modification was set as cysteine modification by iodoacetamide and methionine oxidation was set as variable modification. Search results were evaluated manually and quality MS data confirmed for two peptides and above with E value *P* < 0.05 for each peptide (overall *P* < 0.0025).

Putative protein identifications were subjected to bioinformatic sequence interrogation using Expasy Prosite for motif and sequence analysis (http://www.expasy.org/[accessed January 2015]), specifically interrogating SMART (Simple Modular Architecture Research Tool), Motif search, ELM (Eukaryotic Linear Motif resource), Prosite, Scratch protein predictor and Top pred (Mobyle portal) KEGG (Kyoto encyclopedia of genes and genomes) [47] database searching was also conducted on protein identifications to determine potential related biochemical pathways.

## 3 Results

### 3.1 PPO activity levels and VFA concentrations in wild-type versus mutant genotypes

**Fig. 2:**
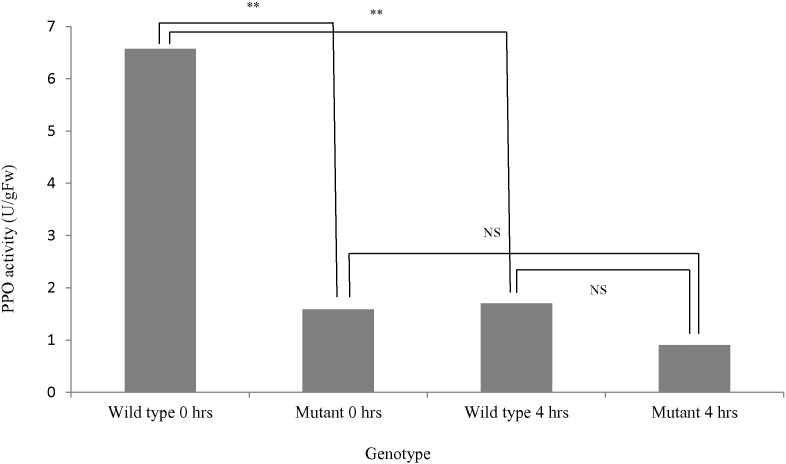
PPO activity (units/g Fw) in boli from wild-type and mutant genotypes at 0 h and after 4 h incubation. Significant differences in PPO activity are denoted by ** where *P* < 0.01, non-significant differences are shown as NS between bars.

Comparison of the active pool of PPO in boli formed from the wild-type red clover plants and the mutant red clover genotype at both 0 and 4 h of anaerobic incubation demonstrated significantly higher activity (*P* < 0.01) in the wild-type (Fig 2). However, the wild-type also showed a significant reduction (*P* < 0.01) in PPO activity post incubation. No significant difference in the level of PPO activity (*P* > 0.05) was determined post incubation for the mutant genotype. Total VFA concentration was unaffected (*P* > 0.05) by the presence of PPO, but was affected by time (*P* < 0.05) with mean values of 51.9 and 157.3 mM for 0 and 4 h respectively, indicative of a normal fermentation.

### 3.2 Comparison of wild-type vs mutant proteomes

**Fig. 3:**
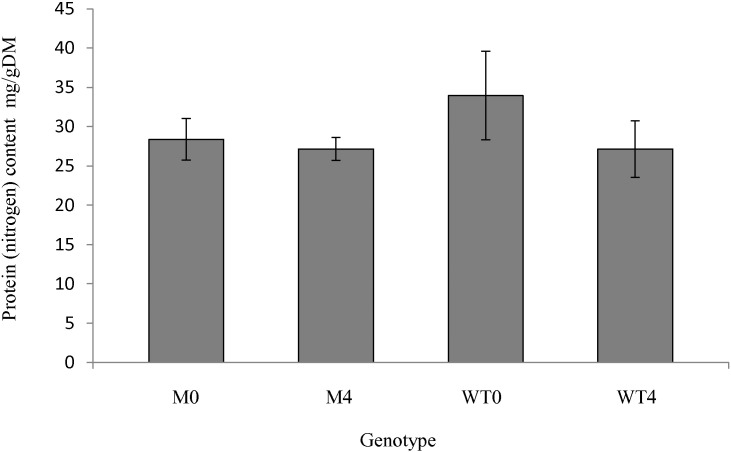
Average total protein (nitrogen) content of boli from wild-type and mutant genotypes at 0 h and after 4 h incubation.

**Fig. 4:**
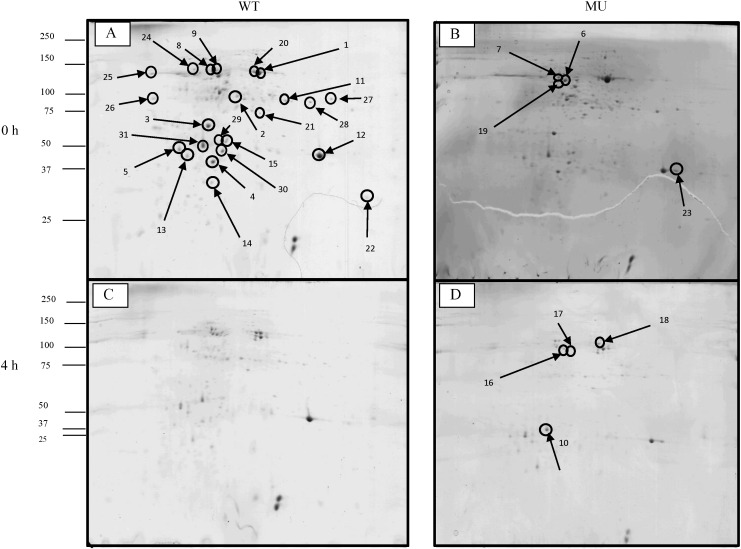
Proteomic profiles of boli formed from wild type and mutant red clover at 0 h and after 4 h incubation. Gels were run on 3–10 non-linear IPG strips, 14% T, 3.3% C SDS-PAGE. Gel images represent gel averages from three biological replicates. Gel A = wild type at 0 h, Gel B = mutant at 0 h, Gel C = wild type at 4 h, Gel D = mutant at 4 h. Circled spots indicate those excised for mass spectrometry.

**Fig. 5:**
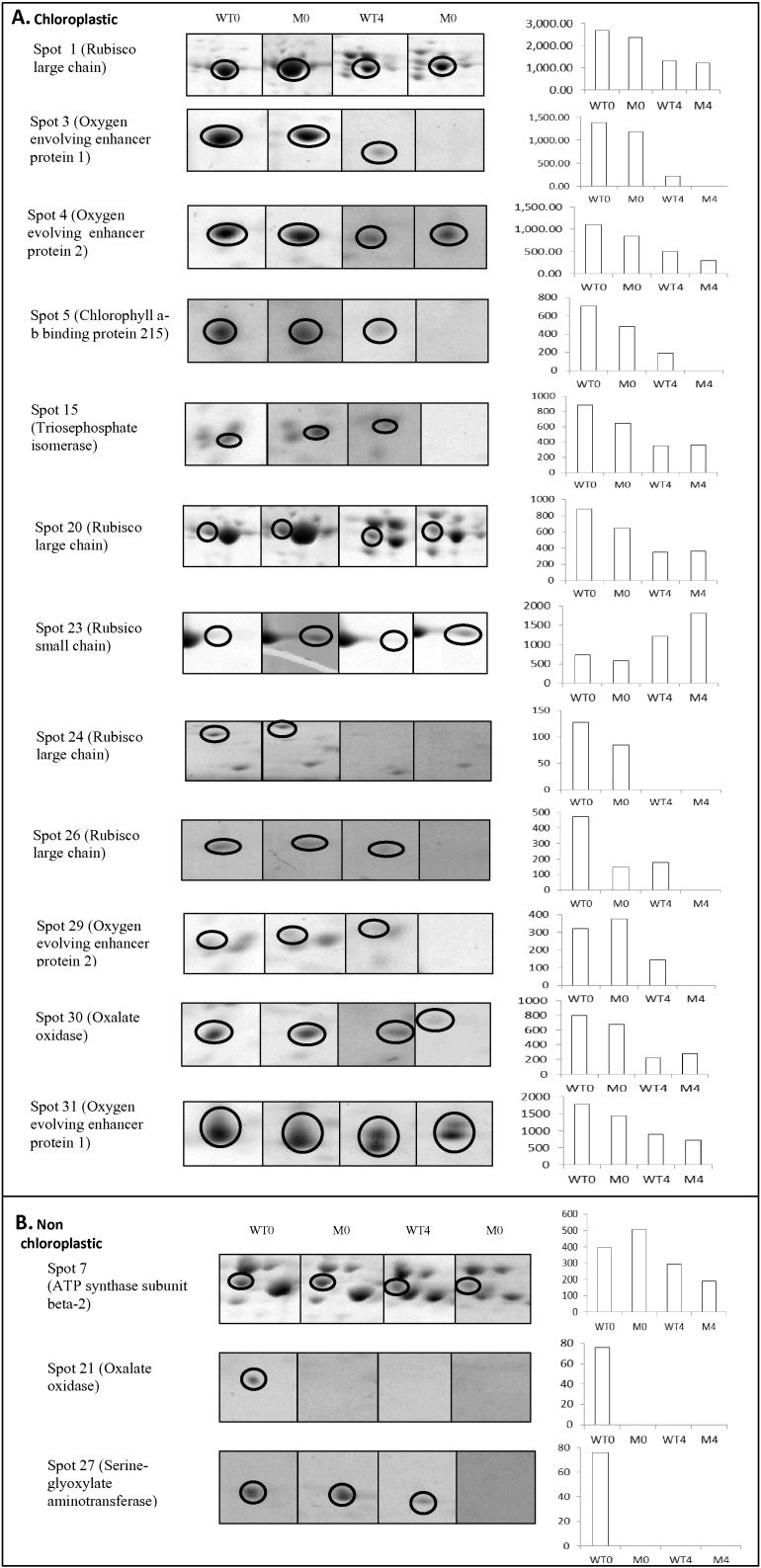
Montage images for wild type 0 and 4 h (WT0, WT4) and mutant 0 and 4 h (M0, M4) average gels and corresponding normalised volume graphs for the top 15 protein spots showing the greatest difference in spot normalisation volume between wild-type and mutant proteomes. Images and graphs for each spot are separated into boxes A and B based on the cellular location of each protein from putative identifications (Fig. 4, Table 1).

In the mutant the 4 h incubation resulted in a decrease in dry matter content from 12.5% to 9% whereas in the wild type the dry matter content was unaffected by incubation (recorded as 8% at both 0 and 4 h). Loss of total nitrogen as a result of the 4 h incubation was observed to be greater in the mutant (26% ± 0.9 SEM) than in the wild-type (55% ± 1.14 SEM) indicating differential loss of protein from mutant and wild type (Fig 3). Two dimensional proteomic analyses were used to specifically determine which proteins were affected by the presence or absence of PPO. The distributions of proteins within the protein profiles were examined and the relative abundance of spots present in 150 μg of protein extract computed to identify protein spots with altered relative abundance according to time or genotype. Reproducible 2D profiles were generated from both wild-type and mutant genotypes; matching between replicate gels averaged over 70% using Progenesis software. Genotype and time-dependent differences in 2D profiles were observed (Fig. 4, Fig. 5, Suppl. Table S1). In the wild-type, incubation for 4 h resulted in no overall change in total spot number (112 spots) of the averaged 2D profile compared with the total spot number of the wild type at 0 h (Fig. 4), but in terms of normalised spot volume 25 of those spots had decreased abundance at 4 h compared with the wild type protein profile at 0 h. In the mutant genotype, 4 h incubation resulted in a decrease in total spot number as compared with the profile observed for the 0 h time point (from 172 average spot numbers at 0 h to a total of 55 average spot numbers at 4 h) with 117 of the spots present at 0 h no longer present on the protein profile at 4 h. Of those spots present in both mutant and wildtype after 4 h incubation, 12 were determined to be of decreased abundance in the mutant profile as compared to the wildtype profile.

### 3.3 Identifications and functional characterisation of differentially abundant proteins

Landmark spots or spots presenting differences in protein abundance (increased, decreased or absent; Fig. 4) were putatively identified (Table 1) following in gel tryptic digestion and subsequent QTOF LCMS/MS analysis. Out of the 31 spots excised for mass spectrometry all were assigned to the kingdom plantae. Of the 31 proteins identified five corresponded to ribulose bisphosphate carboxylase/oxygenase (Rubisco) large subunit and one to the small subunit. Five spots corresponded to oxygen evolving enhancer proteins and five proteins were putatively identified as ATP synthase subunits. The remaining proteins identified within this dataset were fructose bisphosphate, aldolase, oxalate oxidase and chlorophyll a-b binding protein relating to two protein spots each. Spots identified as malate dehydrogenase, glyceraldehyde 3 phosphate dehydrogenase, 50S ribosomal protein, triosephosphate isomerase, sedoheptulose-1,7 bisphosphatase, cytochrome b6-f complex, serine-glyoxylate aminotransferase and apocytochrome all corresponded to a single spot each. Overall, the normalised spot volumes were decreased in the majority of protein spots for both the wild-type and mutant profiles following 4 h incubation (Fig. 4) indicating protein degradation over time. These included the Rubisco large subunit (spots 1,20,24,25 and 26), malate dehydrogenase (spot 2), oxygen enhancer proteins (spot 3, 4, 12, 29 and 31), chlorophyll a-b binding protein 215 (spot 5) and chlorophyll a-b binding protein (spot 13), sedoheptulose 1.7 bisphosphatase (spot 19), oxalate oxidase (spots 21 and 30) and cytochrome B6 f complex iron (spot 22). After 4 h incubation many of these spots were found to be decreased in normalised spot volume in the wildtype and absent from the mutant (Table 1, Fig. 4 and Suppl. Table S1, Fig, S1.). These included ATP synthase beta proteins (spots 6, 7 and 8), 50s ribosomal protein (spot 14), triosephosphate isomerase (spot 15), fructose bisphosphate aldolase (spots 16 and 17) and serine-glyoxylate aminotransferase (spot 27). An increase in normalised spot volume in both the mutant and the wild type was observed post 4 h incubation for three spots (numbers 10, 18 and 23) corresponding to chlorophyll *a*-b binding protein 8, ATP synthase subunit alpha and RUBISCO small subunit respectfully (Fig. 4, Suppl. Fig. S1), indicating their relative preservation within the total protein pool as other proteins were degraded. It has been proposed previously that PPO mediated complexing involves the sulphur amino acids [16]. However, motif searching within the peptide sequences did not reveal any common regions or pattern between those proteins that remained in the wild-type post incubation compared with those that were no longer present in terms of methionine and cysteine content, with and without the inclusion of disulphide bridges. Alternatively, bioinformatic searches indicated that proteins that were preserved in the wild type showed potential phosphoprotein and acetylation modifications (spots 5, 6, 7 and 27), with several proteins having transmembrane domains (5, 6, 7, 14, 16, 17 and 27).

**Fig. 6:**
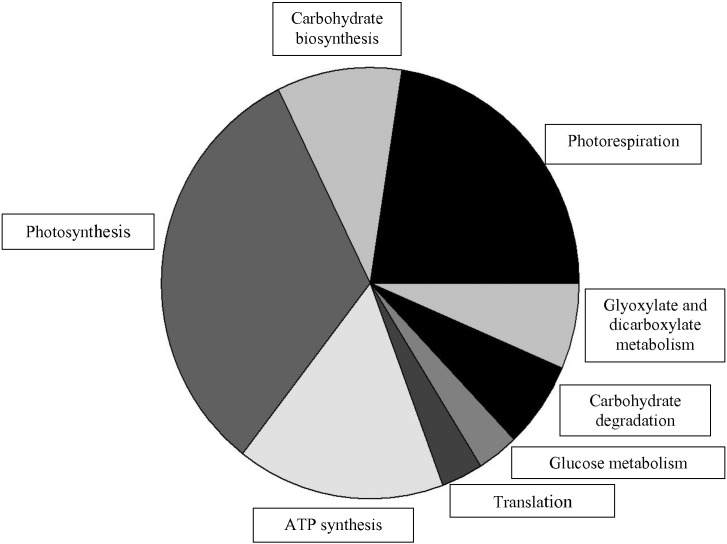
Functional classification of differentially abundant protein identifications from spots excised (Fig. 4). Functional classifications were determined from putative identification descriptions from Uniprot.

Bioinformatic interrogation of protein sequences determined that the majority of spots identified (88%) were associated with the chloroplastic function of the cell (Table 1, Suppl. Table S2), with many of the proteins located within the thylakoid membrane. The remaining proteins were located in either the apoplast or the mitochondria. Variations of normalised spot volumes corresponding to the top 15 most significant spot normalisation volume differences were observed for spots belonging to both the chloroplastic and non-chloroplastic regions of the cell (Fig. 5). Although several proteins were determined to include a transit peptide to the chloroplast region, this was not exclusive to either those deemed preserved in the presence of PPO or degraded. Protein identifications were subjected to KEGG (Kyoto encyclopedia of genes and genomes) (http://www.kegg.jp/kegg/kegg2.html [accessed January 2015]) pathway database analysis, which confirmed that most of the identified proteins (over 50%) were involved in biochemical pathways relating to photosynthesis and photorespiration (Fig. 6). The remaining proteins were involved in ATP synthesis, electron transport, translation, glycolysis, oxidoreductase activity and carbohydrate metabolism (Fig. 6).

## 4 Discussion

The inefficient utilisation of dietary forage protein could potentially be improved by decreasing the extent of protein degradation that occurs within the rumen, and PPO containing forages could be a sustainable mechanism which contributes to achieving this. The subsequent increased flow of non-degraded proteins to the small intestine will improve the nutritional value of the forage feed [31], [48], contributing to overall improved animal productivity. Within the rumen, protein breakdown occurs rapidly during the first 6 h of ingestion [31], with transition from primary to secondary microbial colonisation occurring at 4 h [35]. Therefore, targeting mechanisms to decrease forage protein degradation within this early post-ingestive time frame is important if we are to reduce protein losses from ruminants. Here we have investigated the forage proteomes of a wild-type and a low PPO mutant red clover to determine if the presence of PPO can influence the stability of specific proteins under a fresh forage feeding strategy.

PPO activation is a rapid process. Protein complexing has been suggested to occur in the presence of oxygen during mastication and to continue within the rumen for a limited time. High molecular weight protein complexes have been shown to form in red clover over a 15 min period [5], [27]. The presence of oxygen initiates the formation of covalent bonds of o-quinones to the nucleophilic groups of amino acids [27] and once commenced continues spontaneously [15]. In the present study, the wild-type red clover exhibited higher levels of PPO activity than the low PPO mutant at both 0 and 4 h. Nevertheless, PPO activity decreased in both the wild-type and mutant genotypes over the incubation period, suggesting a time limitation on any potential protective effect of PPO. This correlates with recorded oxygen depletion rates within the rumen, for example [49] determined oxygen levels in grass boluses to be 5.8 mg O_2_/L upon mastication, reducing to 0.05 mg O_2_/L over a period of 10 min, which was further lowered in the presence of ruminal microbes which consume O_2_ [16]. Therefore, it is unlikely that sustained PPO activity occurs in the rumen so early events involving mastication will be important to exploiting the protective potential of PPO containing crops in a fresh forage scenario.

Previous work has determined the inhibition of proteolysis of wild type red clover compared to the low PPO mutant during ensilage [17], [50] and in the simulated rumen PPO has been demonstrated to reduce plant-mediated proteolysis in fresh-fed forages [13], [51]. However, realisation of the potential for PPO to protect protein from proteolysis is likely to depend on the extent of cell damage during mastication as it is this which enables enzyme and substrates to come in to contact. Hence, here we have used a more realistic model to those used previously by using forage down boli captured immediately post mastication. The resulting proteomic differences in protein profiles between the wild-type and low PPO mutant in this study demonstrated the ability of PPO to have a protective effect on the red clover proteins in the fresh forage down bolus, confirming the indications of previous studies. Our results clearly showed that significant degradation of some of the polypeptides contained in the ingested down bolus took place over the 4 h incubation for both wild-type and mutant red clover lines. However, significantly more spots were lost during the 4 h incubation in the low PPO mutant compared to the wild-type, in accordance with the proposed mechanism of protein protection.

The rapid breakdown of plant protein in the rumen is attributed to both rumen microbial activity as well as proteolysis catalysed by plant enzymes [7], [52]. It has been shown that plant protein can be protected from degradation through the presence of PPO activity, mediated through the complexing of plant proteins [12], [36]. While this is generally considered to result in altered structure of the bound proteins, thus making them unsuitable for proteolysis (e.g. by removing access to cleavage site), direct quinone-protease binding will also lead to decreased proteolysis through inactivation of proteases. Although 31 spots were identified as being affected by the presence of PPO (Table 1), these did not include candidate protease targets for PPO-mediated quinone protein binding. The majority of the proteins detected as being affected by the presence/absence of PPO were chloroplastic (Table 1) with relatively few of those identified being located in the cytosol. PPO has previously been shown to be located in the thylakoid membrane of the chloroplast [53], [54], [55] suggesting that in the wild-type red clover these chloroplastic proteins may be subjected to PPO mediated protection to a greater extent due to their proximity to the enzyme and substrates which must either diffuse into this compartment from the vacuole or be present in the chloroplast. A chloroplastic substrate for PPO has been identified in walnut [56] but to date remains to be identified in red clover [57].

PPO induced quinones are highly reactive and have been shown to affect protein physicochemical properties and their susceptibility to degradation [58], [59]. Quinones are strong electrophiles binding to nucleophilic groups of amino acids [60], but exactly how quinone complexes form still remains largely unknown. Sulphur containing amino acids (methionine and cysteine) may act as PPO induced quinone binding sites to form protein bound phenol [16]. It is well established that increasing the content of sulphur-amino acids would increase delivery of what are essential dietary amino acids for ruminants [48], [61] suggesting a possible further role for PPO in protecting these sulphur containing amino acids from metabolism within the rumen [62] thereby making them relatively more available for ruminant nutrition and less prone to wasteful deamination. However, it is unknown whether the acid conditions within the lower gut would be sufficient to reverse the quinone binding of peptides to prevent onset of anti-nutritional effects [63].

In this study, sequence interrogation of red clover proteins showed the presence of a number of methionine and cysteine residues ranging from 2 to 5% of the amino acid content in all proteins excised for identification. Although, no definitive distinction could be observed relating amino acid content and protein degradability, a large proportion of those proteins preserved in the presence of PPO contained methionine at a level of 2% and above. In contrast to red clover, the legume alfalfa is high in protein content but with little PPO activity [3] compared to red clover and has also been shown to be lacking in sulphur containing amino acids [64]. It is not surprising that many proteins were identified here as ribulose bisphosphate, considering that this is the most abundant protein present in plants [65]. However, although the sulphur amino acid content (methionine and cysteine) of these proteins was relatively high (4%) compared to some other proteins identified in this study, the majority appeared to be degraded in the wild-type and mutant profiles to similar extents following incubation.

In conclusion, the results presented here indicate that increasing our ability to exploit PPO mediated protein complexing could be incorporated into strategies to ameliorate losses of dietary protein in general, but could also help with delivery of essential nutrients to enhance animal health by increasing efforts in targeted improvement of PPO containing species such as red clover and cocksfoot.

## Conflict of interest

The authors declare that there is no conflict of interest.

## Figures and Tables

**Table 1:** Spot numbers corresponding to excised spots from gels (Fig. 4) showing normalised spot volumes for the mutant at 0 h (0 M), wild-type and 4 h (4WT) and mutant at 4 h (4 M) compared to the wild type at 0 h. Putative identifications of each spot and corresponding protein locations for each identification are shown together with the accession number and Mascot score. Spots not present (np) are indicated.

Spot no.	Description	Accession	Species	Location	Mascot score	Abundance		
0 M	4WT	4 M
1	Ribulose bisphosphate carboxylase large chain	RBL_BYRCR	*Byrsonima crassifolia*	Chloroplast	481	2686.8	2368.2	350.8
2	Malate dehydrogenase	MDH_PSEM	*Pseudotsuga menziesii*	Mitochondria	156	271.1	546.1	604.6
3	Oxygen-evolving enhancer protein 1	PSBO_PEA	*Pisum sativum*	Chloroplast	415	1377.4	1180.8	214.9
4	Oxygen-evolving enhancer protein 2	PSBP_PEA	*Pisum sativum*	Chloroplast	83	1106.3	850.4	314.5
5	Chlorophyll a-b binding protein 215	CB215_PEA	*Pisum sativum*	Chloroplast	137	711.4	481.4	193.0
6	ATP synthase subunit beta	ATPB_HYANO	*Hyacinthoides non-scripta*	Chloroplast	350	656.6	813.1	652.3
7	ATP synthase subunit beta-2	ATPBN_ARATH	*Arabidopsis thaliana*	Mitochondria	141	397.6	506.2	293.3
8	ATP synthase subunit alpha	ATPA_BUXMI	*Buxus microphylla*	Chloroplast	214	455.2	358.8	588.9
9	ATP synthase subunit alpha	ATPA_BUXMI	*Buxus microphylla*	Chloroplast	163	283.2	313.1	113.7
10	Chlorophyll a-b binding protein 8	CB28_PEA	*Pisum sativum*	Chloroplast	155	319.9	np	np
11	Glyceraldehyde-3-phosphate dehydrogenase A	G3PA_PEA	*Pisum sativum*	Chloroplast	63	400.6	344.7	136.3
12	Oxygen-evolving enhancer protein	PSBP_WHEAT	*Triticum aestivum*	Chloroplast	231	np 4441.1 np		
13	Chlorophyll a-b binding protein	CB23_ORYSI	*Oryza sativa indica group*	Chloroplast	72	526.7	318.7	221.5
14	50s Ribosomal protein L12	RK12_ORYSJ	*Oryza sativa* subsp. *japonica*	Chloroplast	216	279.4	775.2	501.2
15	Triosephosphate isomerase	TPIC_SECCE	*Secale cereale*	Chloroplast	84	374.3	354.5	82.2
16	Fructose-bisphosphate aldolase	ALFC_ORYSJ	*Oryza sativa japonica group*	Chloroplast	360	152.4	174.1	291.7
17	Fructose-bisphosphate aldolase	ALFC_ORYSJ	*Oryza sativa japonica group*	Chloroplast	345	125.3	np	np
18	ATP synthase subunit alpha	ATPA_LOLPR	*Lolium perenne*	Chloroplast	643	np	np	527.8
19	Sedoheptulose-1.7-bisphosphatase	S17P_WHEAT	*Triticum aestivum*	Chloroplast	479	131.5	np	np
20	Ribulose bisphosphate carboxylase large chain	RBL_LOLPR	*Lolium perenne*	Chloroplast	485	887.3	650.9	160.2
21	Oxalate oxidase	GER2_WHEAT	*Triticum aestivum*	Apoplast	108	75.7	np	np
22	Cytochrome b6-f complex iron-sulphur subunit	Q7X9A6	*Triticum aestivum*	Chloroplast	42	334.2	np	np
23	Ribulose bisphosphate carboxylase small chain	RBS_FAGCR	*Fagus crenata*	Chloroplast	28	730.8	581.6	1216.2
24	Ribulose bisphosphate carboxylase large chain	RBL_LOLPR	*Lolium perenne*	Chloroplast	627	458.1	np	np
25	Ribulose bisphosphate carboxylase large chain	RBL_LOLPR	*Lolium perenne*	Chloroplast	627	2686.8	2368.2	350.8
26	Ribulose bisphosphate carboxylase large chain	RBL_LOLPR	*Lolium perenne*	Chloroplast	627	271.1	546.1	604.6
27	Serine–glyoxylate aminotransferase	SGAT_ARATH	*Arabidopsis thaliana*	Apoplast	52	1377.4	1180.8	214.9
28	Apocytochrome	CYF_LOLPR	*Lolium perenne*	Chloroplast	502	1106.3	850.4	314.5
29	Oxygen-evolving enhancer protein 2	PSBP_WHEAT	*Triticum aestivum*	Chloroplast	184	711.4	481.4	193.0
30	Oxalate oxidase	GER2_WHEAT	*Triticum aestivum*	Chloroplast	127	656.6	813.1	652.3
31	Oxygen-evolving enhancer protein 1	PSBO_WHEAT	*Triticum aestivum*	Chloroplast	1684	397.6	506.2	293.3

## Appendix A Supplementary data

Supplementary material Image 2
