# Supplementary material for: The effects of PPO activity on the proteome of ingested red clover and implications for improving the nutrition of grazing cattle
Source: J Proteomics. 2016 Jun 1;141:67–76. doi: 10.1016/j.jprot.2016.04.023 (PMC4881418; doi:10.1016/j.jprot.2016.04.023)
Supplement: Supplementary file 1 — Supplementary material [file mmc1.docx]

|  | **Normalised volumes** | | | | **Significance** | | |  |
| --- | --- | --- | --- | --- | --- | --- | --- | --- |
| **Spot number** | **Wild type 0 h** | **Mutant 0 h** | **Wild type 4 h** | **Mutant 4 h** | **Type** | **Time** | **Type vs Time** | **Regulation** |
| **1** | 2,686.6 | 2,368.2 | 1309.5 | 1210 | 0.956 | **0.035** | 0.647 | Wild-type and mutant at 4 hours more than 2 fold down in abundance compared to wild-type at 0 hours |
| **2** | 377.6 | 152.94 | 256.9 | 196.9 | 0.982 | 0.728 | 0.105 | Mutant at 4 hours and wild type at 4 hours are down in abundance compared to wild type at 0 hours |
| **3** | 1,377.4 | 1,180.8 | 214.9 | *np* | 0.334 | **<.001** | 0.965 | Wild-type at 4 hours is more than 2 fold down in abundance and is not present in mutant at 4 hours compared to wild type at 0 hours |
| **4** | 1,106.3 | 850.4 | 501.2 | 290.7 | 0.278 | **0.025** | 0.911 | Wild-type and mutant at 4 hours are more than 2 fold down in abundance compared to wild-type at 0 hours |
| **5** | 711.4 | 481.4 | 193.0 | *np* | 0.151 | **0.004** | 0.777 | Wild-type at 4 hours is more than 2 fold down in abundance and is not present in mutant at 4 hours compared to wild type at 0 hours |
| **6** | 656.6 | 813.1 | 652.3 | 243.9 | 0.664 | 0.334 | 0.341 | Mutant at 4 hours is more than 2 fold down in abundance compared to wild type at 0 hours |
| **7** | 397.6 | 506.2 | 293.3 | 189.7 | 0.969 | **0.013** | 0.129 | Mutant at 4 hours is more than 2 fold down in abundance compared to wild type at 0 hours |
| **8** | 668.4 | 561.2 | 572.3 | 372 | 0.503 | 0.533 | 0.836 | Wild-type and Mutant at 0 hours down in abundance compared to wild-type and mutant at 4 hours |
| **9** | 331.9 | 236.7 | 134.6 | 242.9 | 0.703 | 0.649 | 0.269 | Wild-type and mutant at 4 hours down in abundance compared to wild-type at 0 hours |
| **10** | 319.9 | *np* | *np* | 733.4 | ~ | ~ | ~ | Mutant at 4 hours is more than 2 fold up regulated compared to wild-type at 0 hours |
| **11** | 400.6 | 344.7 | 277.3 | 221.7 | 0.684 | 0.237 | 0.406 | Wild-type at 4 hours is more than 2 fold down in abundance compared to wild-type at 0 hours |
| **12** | 4,441.1 | 3,856.1 | 5,598.6 | 4,609.9 | 0.346 | 0.258 | 0.804 | Prominent landscape marker spot present on all gels |
| **13** | 526.7 | 318.7 | 221.5 | 185.5 | 0.365 | 0.129 | 0.516 | Wild-type at 4 hours more than 2 fold down in abundance compared to wild type at 0 hours |
| **14** | 279.4 | 775.2 | 501.2 | *np* | 0.994 | 0.426 | 0.175 | Mutant at 0 hours more than 2 fold up in abundance compared to wild-type at 0 hours |
| **15** | 374.3 | 354.5 | 82.2 | *np* | 0.477 | **0.003** | 0.66 | Wild-type at 4 hours is more than 2 fold down in abundance and is not present in mutant at 4 hours compared to wild type at 0 hours |
| **16** | 152.4 | 174.1 | 291.7 | 193.8 | 0.515 | 0.203 | 0.321 | Wild-type at 4 hours 2 fold up in abundance compared to wild-type at 0 hours |
| **17** | 329.6 | 312.8 | 267.1 | 171.3 | 0.457 | 0.194 | 0.598 | Mutant at 4 hours more than 2 fold down in abundance compared to wild-type at 0 hours |
| **18** | *np* | *np* | 527.8 | 438.5 | ~ | ~ | ~ | Present in wild-type and mutant at 4 hours, not present at 0 hours |
| **19** | 234.4 | 310.8 | *np* | *np* | ~ | ~ | ~ | Only present in the wild-type at 0 hours |
| **20** | 887.3 | 650.9 | 352.6 | 363.3 | 0.933 | **0.05** | 0.874 | Wild-type and mutant at 4 hours more than 2 fold down in abundance compared to wild-type at 0 hours |
| **21** | 75.7 | *np* | *np* | *np* | ~ | ~ | ~ | Only present in the wild-type at 0 hours |
| **22** | 334.2 | *np* | *np* | *np* | ~ | ~ | ~ | Only present in the wild-type at 0 hours |
| **23** | 730.8 | 581.6 | 1216.2 | 1,814.2 | 0.725 | 0.492 | 0.541 | Up in abundance at 4 hours in wild-type and mutant, more than 2 fold up in abundance in the mutant after 4 hours |
| **24** | 128 | 85 | *np* | *np* | 0.467 | **0.009** | 0.467 | Not present in the wild-type or mutant at 4 hours |
| **25** | 717.6 | 720.3 | 463.9 | 441.6 | 0.924 | 0.189 | 0.934 | Mutant at 0 hours and wild-type and mutant at 4 hours more than 2 fold down in abundance compared to the wild-type at 0 hours |
| **26** | 470 | 149 | 176 | *np* | **<0.001** | **<0.001** | **0.026** | Wild-type at 4 hours and mutant at 0 hours more than 2 fold down in abundance compared to wild-type at 0 hours, not present in the mutant at 4 hours |
| **27** | 147 | 59 | 188 | *np* | 0.073 | 0.892 | 0.458 | Not present in the mutant at 4 hours |
| **28** | 172 | 348 | 266 | 140 | 0.853 | 0.680 | 0.292 | Mutant at 4 hours more than 2 fold down in abundance compared to mutant at 0 hours |
| **29** | 320 | 377 | 145 | *np* | 0.580 | **0.014** | 0.232 | Wild-type at 4 hours more than 2 fold down in abundance compared to wild-type at 0 hours, not present in mutant at 4 hours |
| **30** | 798 | 678 | 219.7 | 274.8 | 0.602 | 0.067 | 0.579 | Wild-type at 4 hours more than 2 fold down in abundance compared to wild-type at 0 hours |
| **31** | 1776 | 1440 | 907 | 732 | 0.330 | **0.014** | 0.751 | Wild-type and mutant at 4 hours more than 2 fold down in abundance compared to wild-type at 0 hours |

Supplementary Material

Table S1. Normalised spot volumes and significance values (P<0.05) dependent on phenotype (type), time and interaction (type vs time) for each gel spot excised and regulation description when comparing each gel type to the wild type at 0 hours (+/- 2 fold abundance).


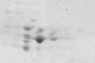

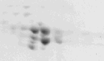


15

13


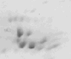

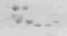


16

12


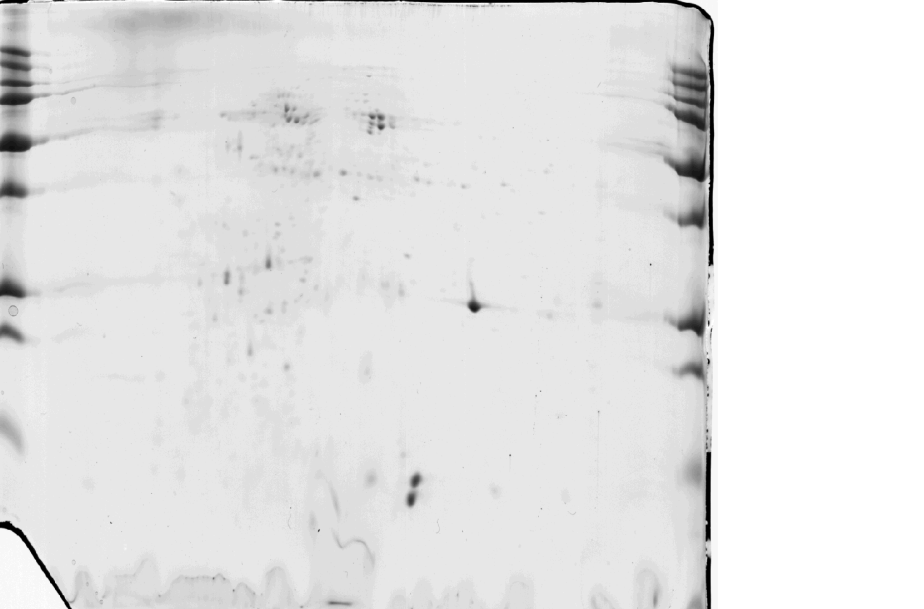

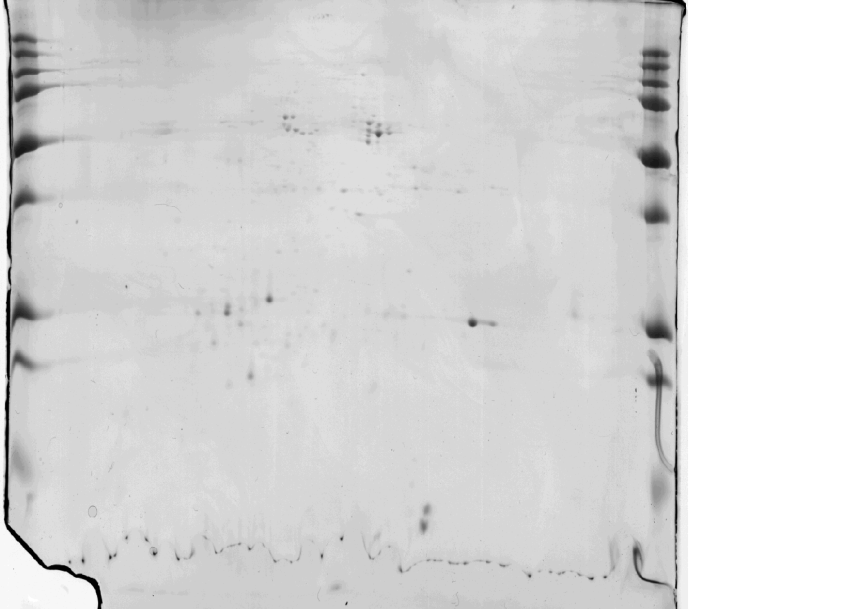


B

A


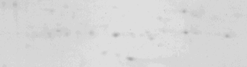

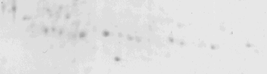


27

10


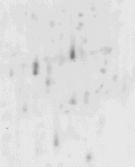


26


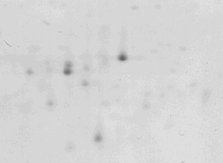


7

Figure S1. Comparison of Wild type (A) and Mutant (B) protein profiles after 4 hours incubation. With highlighted sections showing differences in gel regions between phenotype and spot count for each region.

| **Spot Number** | **Accession** | **Description** | **Species** | **Location** | **Peptide Sequence** | **Mascot score** | **Sequence coverage (%)** |
| --- | --- | --- | --- | --- | --- | --- | --- |
| **1** | RBL_BYRCR | Ribulose bisphosphate carboxylase large chain | *Byrsonima crassifolia* | Chloroplast | TFQGPPHGIQVER  EITLGFVDLLR  DNGLLLHIHR | 481 | 36 |
| **2** | MDH_PSEM | Malate dehydrogenase | *Pseudotsuga menziesii* | Mitochondria | LFGVTTLDVVR  DDLFNINAGIVK | 156 | 100 |
| **3** | PSBO_PEA | Oxygen-evolving enhancer protein 1, | *Pisum sativum* | chloroplast | GASTGYDNAVALPAGGR  DGIDYAAVTVQLPGGER  RLTFDEIQSK | 415 | 19 |
| **4** | PSBP_PEA | Oxygen-evolving enhancer protein 2 | *Pisum sativum* | chloroplast | EFPGQVLR  EFPGQVLR-Pyro-glu | 83 | 15 |
| **5** | CB215_PEA | Chlorophyll a-b binding protein 215 | *Pisum sativum* | chloroplast | SAPESIWYGPDRPK  ELEVIHSR  FGEAVWFK | 137 | 11 |
| **6** | ATPB_HYANO | ATP synthase subunit beta | *Hyacinthoides non-scripta* | chloroplast | AHGGVSVFGGVGER  IFNVLGEPIDNLGPVDTR  VALVYGQMNEPGAR | 350 | 37 |
| **7** | ATPBN_ARATH | ATP synthase subunit beta-2 | *Arabidopsis thaliana* | mitochondria | AHGGFSVFAGVGER  FTQANSEVSALLGR | 141 | 11 |
| **8** | ATPA_BUXMI | ATP synthase subunit alpha | *Buxus microphylla* | chloroplast | IAQIPVSEGYLGR  LIESPAPGIISR EAYPGDVFYLHSR | 214 | 9 |
| **9** | ATPA_BUXMI | ATP synthase subunit alpha | *Buxus microphylla* | chloroplast | IAQIPVSEGYLGR  LIESPAPGIISR  EAYPGDVFYLHSR | 163 | 12 |
| **10** | CB28_PEA | Chlorophyll a-b binding protein 8 | *Pisum sativum* | chloroplast | VASSGSPWYGPDR  KVASSGSPWYGPDR | 155 | 11 |
| **11** | G3PA_PEA | Glyceraldehyde-3-phosphate dehydrogenase A | *Pisum sativum* | chloroplast | TFAEEVNEAFR | 63 | 6 |
| **12** | PSBP_WHEAT | Oxygen-evolving enhancer protein | *Triticum aestivum* | chloroplast | EFPGQVLR  QYYSITVLTR  HQLITATVADGK  KFVENAAGSFSVA | 231 | 16 |
| **13** | CB23_ORYSI | Chlorophyll a-b binding protein | *Oryza sativa indica group* | chloroplast | ELEVIHSR  VGGGPLGEGLDK | 72 | 7 |
| **14** | RK12­_ORYSJ | 50s Ribosomal protein L12 | *Oryza sativa subsp.japonica* | chloroplast | VLELGDAIAGLTLEEAR  TEFDVVIEEVPSSAR | 216 | 16 |
| **15** | TPIC_SECCE | Triosephosphate isomerase | *Secale cereale* | chloroplast | HVIGEDDEFIGK  VASPEQAQEVHAAVR  GPDFATICNSVTSK | 84 | 13 |
| **16** | ALFC_ORYSJ | Fructose-bisphosphate aldolase | *Oryza sativa japonica group* | chloroplast | GILAMDESNATCGK  LASIGLENTEANR  EAAYYQQGAR  TVVSIPNGPSELAVK  ALQNTCLK  ANSLAQLGKYTSDGEAAEAK | 360 | 20 |
| **17** | ALFC_ORYSJ | Fructose-bisphosphate aldolase | *Oryza sativa japonica group* | chloroplast | GILAMDESNATCGK  LASIGLENTEANR  EAAYYQQGAR  TVVSIPNGPSELAVK  ALQNTCLK  ANSLAQLGKYTSDGEAAEAK | 345 | 20 |
| **18** | ATPA_LOLPR | ATP synthase subunit alpha | *Lolium perenne* | chloroplast | KVGIENIGRVVQVGDGIAR  GIALNLESK  IAQIPVSEAYLGR  GEIIASESRLIESPAPSIISR  TAVATDTILNQK  HTLIIYDDLSK  EAYPGDVFYLHSR  TSQNQLAR  GYLDSLEIEQVNKFLDDLRK  DTKPQFQEILSSSK  EAIQEQLER | 643 | 29 |
| **19** | S17P_WHEAT | Sedoheptulose-1,7-bisphosphatase | *Triticum aestivum* | chloroplast | LLICMGEAMR  TASCGGTACVNSFGDEQLAVDMLADK  LTGVTGGDQVAAAMGIYGPR  DCPGTHEFLLLDEGK  MFSPGNLRATFDNPDYDK  YTGGMVPDVNQIIVK  GIFTNVTSPTAK  FEETLYGSSR | 479 | 32 |
| **20** | RBL_LOLPR | Ribulose bisphosphate carboxylase large chain | *Lolium perenne* | chloroplast | ASVGFQAGVK  LTYYTPEYETKDTDILAAFR  TFQGPPHGIQVER  YGRPLLGCTIKPK  ACYECLRGGLDFTKDDENVNSQPFMR  FVFCAEALYKAQAETGEIKGHYLNATAGTCEEMIK  DNGLLLHIHRAMHAVIDR  MSGGDHIHSGTVVGK  EMTLGFVDLLR  FEFEPVDTIDN | 485 | 36 |
| **21** | GER2_WHEAT | Oxalate oxidase | *Triticum aestivum* | Apoplast | VDFAPGGTNPP HIHPR  GELLVG ILGSLDSGNK  AGETFLIPR | 108 | 18 |
| **22** | Q7X9A6 | Cytochrome b6-f complex iron-sulfur subunit | *Triticum aestivum* | chloroplast | (K)FLCPCHGSQYNNQGK(V)  (K)GDPTYLVVESDK(T) | 42 | 12 |
| **23** | RBS_FAGCR | Ribulose bisphosphate carboxylase small chain | *Fagus crenata* | chloroplast |  | 28 | 28 |
| **24** | RBL_LOLPR | Ribulose bisphosphate carboxylase large chain | *Lolium perenne* | chloroplast | ASVGFQAGVK  LTYYTPEYETKDTDILAAFR  TFQGPPHGIQVER  YGRPLLGCTIKPK  ACYECLRGGLDFTKDDENVNSQPFMR  FVFCAEALYKAQAETGEIKGHYLNATAGTCEEMIK  DNGLLLHIHRAMHAVIDR  NHGMHFR  MSGGDHIHSGTVVGK  EMTLGFVDLLR  FEFEPVDTIDN | 627 | 37 |
| **25** | RBL_LOLPR | Ribulose bisphosphate carboxylase large chain | *Lolium perenne* | chloroplast | ASVGFQAGVK  LTYYTPEYETKDTDILAAFR  TFQGPPHGIQVER  YGRPLLGCTIKPK  ACYECLRGGLDFTKDDENVNSQPFMR  FVFCAEALYKAQAETGEIKGHYLNATAGTCEEMIK  DNGLLLHIHRAMHAVIDR  NHGMHFR  MSGGDHIHSGTVVGK  EMTLGFVDLLR  FEFEPVDTIDN | 627 | 37 |
| **26** | RBL_LOLPR | Ribulose bisphosphate carboxylase large chain | *Lolium perenne* | chloroplast | ASVGFQAGVK  LTYYTPEYETKDTDILAAFR  TFQGPPHGIQVER  YGRPLLGCTIKPK  ACYECLRGGLDFTKDDENVNSQPFMR  FVFCAEALYKAQAETGEIKGHYLNATAGTCEEMIK  DNGLLLHIHRAMHAVIDR  NHGMHFR  MSGGDHIHSGTVVGK  EMTLGFVDLLR  FEFEPVDTIDN | 627 | 37 |
| **27** | SGAT_ARATH | Serine--glyoxylate aminotransferase | *Arabidopsis thaliana* | Apoplast | **YNLSLGLGLNK** | 52 | 2 |
| **28** | CYF_LOLPR | Apocytochrome | *Lolium perenne* | chloroplast | IPYDMQLK  KGGLNVGAVLILPEGFELAPPDR IGNLSFQSYRPDKKNILVIGPVPGKKYSE IVFPILSPDPATK  **YPIYVGGNR**  GQIYPDGSKSNNTVYNATSTGIVK  EKGGYEISIVDASDGRQVIDTIPPGPELLVSEGESIK  VQLYEMNF | 502 | 47 |
| **29** | PSBP_WHEAT | Oxygen-evolving enhancer protein 2 | *Triticum aestivum* | chloroplast | EREFPGQVLR  QYYSITVLTR  HQLITATVADGK  KFVENAAGSFSVA | 184 | 16 |
| **30** | GER2_WHEAT | Oxalate oxidase | *Triticum aestivum* | chloroplast | VDFAPGGTNPPHIHPRATEIGIVMK  AGETFLIPR | 127 | 15 |
| **31** | PSBO_WHEAT | Oxygen-evolving enhancer protein 1 | *Triticum aestivum* | chloroplast | RLTFDEIQSKT  DGIDYAAVTVQLPGGER  GDEEELAKENVKNASSSTGNITLSVTK | 1684 | 16 |

Table S2. Proteins identified by MALDI TOF/TOF mass spectrometry. Gel plugs (fig.1) were excised, trypsin digested and analysed by MALDI TOF/TOF MS. The MS data was queried using the Mascot database.

Table 2. Proteins identified by MALDI TOF/TOF mass spectrometry. Gel plugs (fig.1) were excised, trypsin digested and analysed by MALDI TOF/TOF MS. The MS data was queried using the Mascot database.
